# *Cronobacter* spp. in Common Breast Milk Substitutes, Bogotá, Colombia

**DOI:** 10.3201/eid2410.172021

**Published:** 2018-10

**Authors:** Maria del Rocío Morato-Rodríguez, Daniel Velandia-Rodríguez, Sandra Castañeda, Milton Crosby, Herbert Vera

**Affiliations:** Secretaria Distrital de Salud de Bogotá, Bogotá, Colombia (M. del Rocío Morato-Rodríguez, D. Velandia-Rodríguez, S. Castañeda, H. Vera);; Universidad Nacional de Colombia, Bogotá (M. Crosby)

**Keywords:** *Cronobacter*, infant formulas, zea mays, musa, bacteria, contamination, foodborne infections, breast milk substitutes, Bogotá, Colombia

## Abstract

In Bogotá, Colombia, a large number of babies are fed with breast milk substitutes made from corn and plantain starch. We found 34.3% of tested samples to be contaminated with *Cronobacter* spp.; *C. sakazakii* was the most recovered species. Our findings underscore the risk for contamination of breast milk substitutes.

*Cronobacter* spp. is a group of emerging foodborne opportunistic pathogenic microorganisms that can cause deadly disease in neonates, children, older adults, and immunocompromised persons, including meningitis, septicemia, and necrotizing enterocolitis in neonates and infants (*1*). The presence of *Cronobacter* spp. is widespread in dry foods. Occurrence is higher in infant milk formula (IMF) because some species of *Cronobacter* are able to tolerate different types of stress in IMF; for example, *C. sakazakii* is able to survive up to 2 years in IMF, which increases the possibility for baby foods to become reservoirs, given that milk increases the cultivability and recovery of *C. sakazakii* in dry environments (*2*).

In Colombia, according to the 2010 Nutritional Situation Survey (ENSIN, for its acronym in Spanish), breast-feeding supplementation is gradually made from birth, starting at 27% in the first months until 76% at 9 months of age (*3*). These data suggest that a large number of children can start the intake of supplementary foods, most likely in the form of bottle-feeding (*3*). IMFs are not sterile products; pathogenic microorganisms such as *Salmonella* spp. and *Cronobacter* spp. can be recovered from them (*4*).

At the local level in Colombia, the most common IMF substitutes used are corn and plantain starches. The main reason for their use is the high cost of imported foods (*5*). The microbiologic quality of these foods is important because they are given to breast-fed infants or children <1 year of age, who are vulnerable to infections. However, unlike IMFs, little attention has been paid to the contamination of IMF substitutes with *Cronobacter* spp.

## The Study

We collected information on starch brands and consumption preferences from the stores where starch products were sold. We sampled city districts with the highest frequency of consumption (i.e., >1×/wk). The sampling was distributed per percentage weight according to city district and starch composition. We collected 36 samples of corn starch, 53 samples of plaintain starch, and 13 samples of other starches (N = 102 samples)

According to the evaluation conducted after enactment of the current baby food regulations in Colombia (*6*), product noncompliance was most often attributable to the presence of coliforms, which were detected in 23.5% (24/102) of samples. Coliforms were detected in 50% (12/24) of plantain starch samples, 12.5% (3/24) of other starch samples, and 0% (0/24) of corn starch samples. For the detection of *Cronobacter* spp., we used the ISO 22964:2006 method, by which we were able to recover isolates from 35 (34.3%) samples. However, recovery was already improved in the new ISO 22964:2017 version, which is desirable because *Cronobacter* spp. have been found at levels of <1 UFC/100 g of IMFs (*7*).

**Figure 1:**
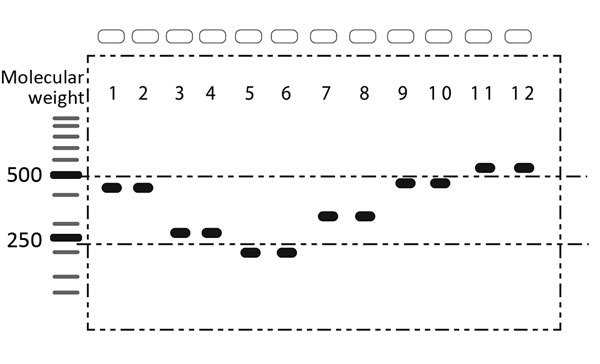
Individual evaluation of multiplex PCR primers used to differentiate 6 species of *Cronobacter* spp. in a study of *Cronobacter* in common breast milk substitutes, Bogotá, Colombia, 2016. Lanes were used as guides for calculating the molecular weight of each band.

We differentiated *Cronobacter* spp. species by using PCR *cgcA* enzyme (*8*) (Platinum Blue PCR Super Mix; Invitrogen, Carlsbad, CA, USA). We then improved specificity and reduced the amplification of nonspecified fragments (Figure 1).

**Figure 2:**
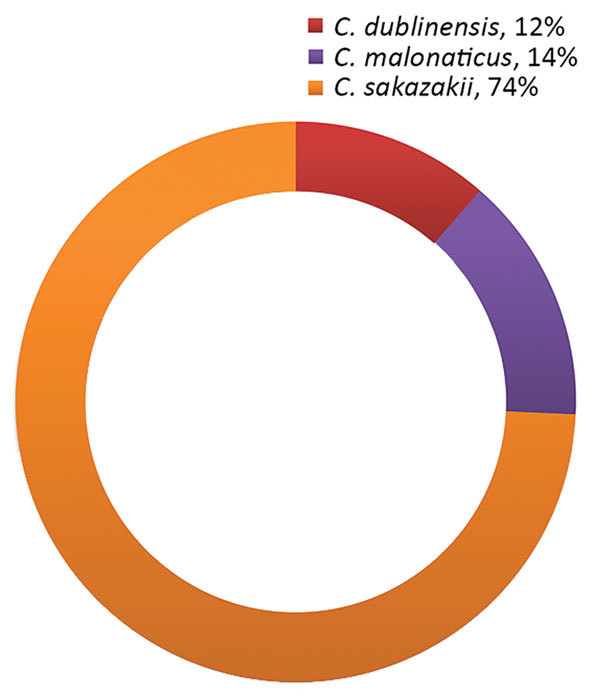
Species of C*ronobacter* recovered from corn, plantain, and other starches in a study of *Cronobacter* spp. in common breast milk substitutes, Bogotá, Colombia, 2016.

Of the 102 samples, 34.3% (35/102) were positive for *Cronobacter* spp. (26.5% [27/35] of plantain starch samples, 7.8% [8/35] of other starch samples, and 0% of corn starch samples). The *Cronobacter* species identified were *C. sakazakii* (74% [26/35] of isolates), *C. malonaticus* (14% [5/35] of isolates), and *C. dublinensis* (11% [4/35] of isolates) (Figure 2).

The 34.3% *Cronobacter* spp. prevalence was similar to that reported in plant derivatives (e.g., 20.1% [*9*] and 31.3% [*10*]) and in cereals (63%) (*11*). Prevalence in processed and other foods has ranged from 3% to 30% (*12*). Although the greatest number of positive isolates was recovered from plantain starches, no information on *Cronobacter* spp. in plantain matrixes is available.

## Conclusions

From the point of view of microbiologic quality, the presence of coliform bacteria and pathogens shows the risk of contamination of IMF substitutes. For *Cronobacter* spp., the international standard is a total absence in IMF (*13*). The most recovered species in the samples we tested was *C.* *sakazakii*, which is the species associated with the highest number of disease cases reported in neonates and whose sequence type 4 is already known to be associated with severe cases of meningitis (*14*). The PCR method we used enabled greater coverage compared with the traditional method because the identification of the species is confirmed in a single analytical run, thus speeding up the subsequent public health response and providing valuable information for epidemiologic surveillance.

In Bogotá, no cases of foodborne diseases related to the presence of *Cronobacter* spp. have been reported. However, *Cronobacter* are not subject to mandatory re-porting in Colombia because no active surveillance of this pathogen exists, and the medical community lacks information about the pathogen. It is important to char-acterize and document the presence of *Cronobacter* spp. in different foods to assess the risk to which children <1 year of age and breast-fed infants are exposed and to identify the connection between diseases like meningitis and the consumption of contaminated baby foods produced locally. Some measures have been proposed to mitigate this risk. It is vital to indicate on the starch packaging that starch must be reconstituted in water at >70°C for >1 minute before consumption, and starch leftovers must be refrigerated to minimize the risk for contamination with *Cronobacter* spp. (*15*). In addition, the World Health Organization–Codex Alimentarius Code of Hygiene Practices provides guidelines for governments, industries, and consumers to support and teach caregivers about safe preparation of starches (*13*). Our findings must lead to an in-depth review of the current regulations for baby foods in Colombia and a modification of Bogotá’s System of Inspection, Surveillance, and Control so that this system can be more preventive and responsive in ensuring the health of citizens.
